# Interactions and Cold Collisions of AlF in the Ground and Excited Electronic States with He

**DOI:** 10.1021/acs.jpca.5c02533

**Published:** 2025-09-02

**Authors:** Sangami Ganesan-Santhi, Matthew D. Frye, Marcin Gronowski, Michał Tomza

**Affiliations:** Faculty of Physics, University of Warsaw, Pasteura 5, 02-093 Warsaw, Poland

## Abstract

Aluminum monofluoride (AlF) is a promising candidate for laser cooling and the production of dense ultracold molecular gases, thanks to its relatively high chemical stability and diagonal Franck–Condon factors. In this study, we examine the interactions and collisions of AlF in its *X* ^1^Σ^+^, *a* ^3^Π, and *A* ^1^Π electronic states with ground-state He using state-of-the-art ab initio quantum chemistry techniques. We construct accurate potential energy surfaces (PESs) employing either the explicitly correlated coupled-cluster CCSD­(T)-F12 method augmented by the CCSDT correction or the multireference configuration-interaction method for higher-excited electronic states. Subsequently, we employ these PESs in coupled-channel calculations to determine the scattering cross sections for AlF + He collisions and bound states of the complex. We estimate the uncertainty of the calculated PESs and apply it to assess the uncertainty of the scattering results. We find a relatively low sensitivity of the cross sections to the variation of the PESs, but the positions of shape resonances remain uncertain. The present results are relevant for further improvements and optimizations of buffer-gas cooling of AlF molecules.

## Graphical abstract

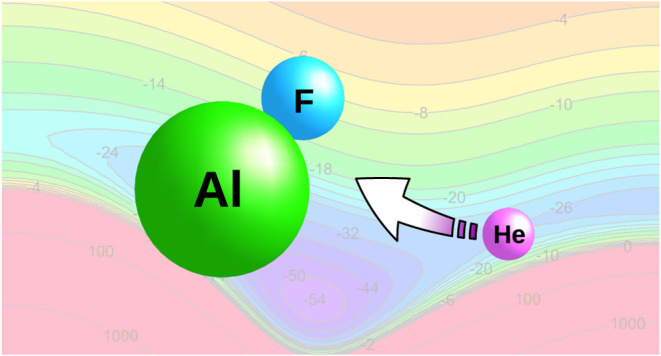

## 1 Introduction

Ultracold systems are an excellent platform for various experiments ranging from controlled chemical reactions^1^ and precision measurements^2^ to quantum simulation of many-body physics^3^ and quantum information processing.^4^ Research into cold and ultracold molecules experienced a significant surge following the first successful production of an ultracold gas of molecules in its absolute ground state.^5^ The complex architecture of energy states within molecules, which comprise electronic, vibrational, rotational, fine, and hyperfine degrees of freedom,^6^ provides opportunities for precise control and manipulation of both internal and translational molecular motion.^7^ This capability facilitates comprehensive explorations of chemical interactions, spectroscopy, and fundamental physics.^4^

Ultracold molecules can be produced by two broad strategies. The first involves photo- or magneto-association of precooled atoms at ultralow temperatures,^8,9^ usually followed by optical transfer to a deeply bound state.^10^ The alternative strategy involves direct cooling of molecules themselves from higher temperatures. Buffer gas cooling^11^ is a direct cooling method in which the translational and internal energies are dissipated through collisions with cold, inert gas atoms, usually helium. This typically serves as one of the initial cooling steps since it is generally effective at temperatures down to a few hundred millikelvin^12^ but can produce high densities in preparation for a further cooling stage to ultracold temperatures. Typically, a molecule in buffer-cell reaches steady-state temperature within milliseconds,^11^ however modern buffer-gas beams can have extraction times as short as 100 μs.^13^ It is therefore important to have sufficiently large cross sections for thermalization of both translational degrees of freedom and internal degrees of freedom, i.e., rotational relaxation; by contrast, buffer gas cooling of molecules trapped in a static magnetic or electric traps are instead typically limited by losses due to spin-changing collisions removing molecules from trappable states. Laser cooling^14^ is another direct cooling method and is frequently used as a second-stage cooling method for molecules, paired with buffer gas cooling. In this process, the directional absorption of photons results in slowing down molecules. This technique requires nearly closed optical cycling and is ubiquitous in atom cooling, but it has also been extended to a particular class of molecules with highly diagonal Franck–Condon factors.^14,15^

The aluminum fluoride (AlF) molecule is an ideal candidate for cooling to ultracold temperatures.^16,17^ This is primarily due to its highly diagonal Franck–Condon factors, which are considered essential for laser cooling.^18,19^ Another advantage of AlF is its chemical stability due to its relatively high binding energy (about 7 eV). As the binding energy of simple monofluorides grows, the efficiency of the reaction of the fluorinating reagent with hot ablated metal also increases, as was demonstrated by theoretical computations for AlF and CaF.^20^ A comparison of buffer-gas-cooled beams of four monofluorides showed that the one containing AlF was an order of magnitude brighter than others due to this increased stability relative to MgF, CaF, and YbF.^21^ New accurate spectroscopic studies of AlF in molecular beams have been recently reported,^18,22,23^ optical cyling has been demonstrated,^16^ and very recently the first MOT of AlF has been achieved.^24^ Once cooled, its relatively large dipole moment of 1.5 D makes it a promising candidate for studying numerous types of dipolar physics.

So far, all experiments have targeted ground electronic state of AlF, and buffer gas cooling has been considered theoretically by Karra et al.^17^ for this state. However, the first excited state of AlF (*a* ^3^Π) is metastable with radiative lifetimes of 1.89(15) ms for its *a* ^3^Π_1_ component,^18,23^ and significantly longer for the *a* ^3^Π_0,2_ components (likely one to 2 orders of magnitude longer, analogous to similar states in CO^25^). These states are likely to be formed in the laser ablation processes used to form the molecules experimentally, or may be populated directly spectroscopically, and are therefore also of great interest. These states may be suitable for buffer-gas cooling, since their lifetimes are long compared to typical time scales for extraction from modern buffer-gas cells. Depending on the true lifetimes, trapping in a magnetic trap and further experiments on this metastable state may be possible. Additionally, 3-body recombination in a buffer gas cell may lead to the formation of triatomic van der Waals molecules AlF–He,^26–28^ which may both affect laser-cooling of AlF and be of interest in their own right. Bound-state calculations are required to understand such molecules in both ground and excited states.

Before AlF became an object of research at ultralow temperatures, the discovery of AlF in a protoplanetary circumstellar envelope sparked astronomers’ interest in this molecule.^29^ For this reason, the first potential energy surface of the ground-state AlF + He complex was reported.^30^ This potential exhibited one minimum only with the well depth of about 24 cm^–1^. Newer studies^17^ suggested that this potential is shallower by about 2 cm^–1^.

In this work, we aim to investigate the prospects for buffer-gas cooling of AlF molecules. As a theoretical foundation, we study in detail the interactions between the AlF molecule in its two lowest electronic states (*X* ^1^Σ^+^, *a* ^3^Π) and ground-state He using accurate ab initio quantum chemistry methods. We compute two-dimensional potential energy surfaces for the five lowest electronic states of the AlF + He complex. We evaluate the accuracy of our calculations by analyzing the convergence with the wave function quality and basis set size and the impact of the relativistic effects. Next, we employ the electronic structure data in coupled-channel scattering calculations of thermalization and inelastic scattering cross sections and their implications for buffer gas cooling. Additionally, we calculate the potential energy for interaction between the AlF molecule in its lowest excited singlet electronic states (*A* ^1^Π) and ground-state helium, which may guide future electronic absorption and laser-induced fluorescence spectroscopy experiments on AlF + He.

The plan of the paper is as follows. The theory behind the construction of the potential energy surface and a brief account of collision theory are discussed in Section 2. Potential energy surfaces and scattering results are presented in Section 3. We conclude our work in Section 4.

## 2 Methods

The *X* ^1^Σ^+^, *a* ^3^Π, and *A* ^1^Π electronic states of AlF upon interaction with ground-state He correspond to the following electronic states of the interacting complex: *X* ^1^ *A*′, *a* ^3^ *A*″, *b* ^3^ *A*′, *A* ^1^ *A*′, and *B* ^1^ *A*″, under the *C*_s_ point group, as collected in Table 1. Schematically, the *a* ^3^Π and *A* ^1^Π AlF states are both obtained by σ → π* excitation from the *X* ^1^Σ^+^ state; due to the strongly ionic nature of the molecule, this largely has the character of an *s* → *p* excitation on Al^+^. When the He approaches, the two potentials are formed with the π* excitation in the triatomic plane (*A*′) and normal to the plane (*A*″). We use advanced ab initio quantum-chemical methods to describe all the mentioned electronic states of AlF + He. Our calculations are based on the Born–Oppenheimer approximation, in which a separate potential energy surface is defined for each electronic state.

**Table 1:** Relation and Symmetry of the Electronic States of the AlF + He Complex to the Electronic States of Interacting AlF and He

AlF	He	AlF + He
*X* ^1^Σ^+^	^1^ *S*	*X* ^1^ *A*′
*a* ^3^Π	^1^ *S*	*b* ^3^ *A*′, *a* ^3^ *A*″
*A* ^1^Π	^1^ *S*	*A* ^1^ *A*′, *B* ^1^ *A*″

### 2.1 Ab Initio Electronic Structure Methods

The AlF molecule is considered as a rigid rotor with a fixed bond length *r*_AlF_. The Jacobi coordinates *R* and θ are used to describe the orientation of the molecule and the atom. *R* is the distance between the helium atom and the center of mass (c.m.) of the molecule and θ is the angle between the molecular axis and vector from c.m. to He (θ = 0° and θ = 180° correspond to the linear HeAlF and AlFHe arrangements, respectively). The coordinates are presented in Figure 1. We use the vibrationally averaged value of the bond length *r*_AlF_ for the corresponding electronic states^31,32^ (3.136 bohr for *X* ^1^Σ^+^, 3.124 bohr for *a* ^3^Π, and 3.126 bohr for *A* ^1^Π). The interaction potential, *V*_int_, depends on both *R* and θ. We obtain *V*_int_(*R*,θ) by using the supermolecular method

1 $$V_{\mathrm{int}}\left(R,\theta \right)=E_{\mathrm{AlF}+\mathrm{He}}\left(R,\theta \right)-E_{\mathrm{AlF}}\left(R,\theta \right)-E_{\mathrm{He}}\left(R,\theta \right)$$

where *E*_AlF+He_ is the total energy of the complex in the dimer basis set, while *E*_AlF_ and *E*_He_ are the energies of the monomers in the dimer basis set. The basis set superposition error is corrected using the counterpoise correction,^33^ where the monomer energies are also calculated in the same basis set as that of the whole complex.

**Figure 1:**
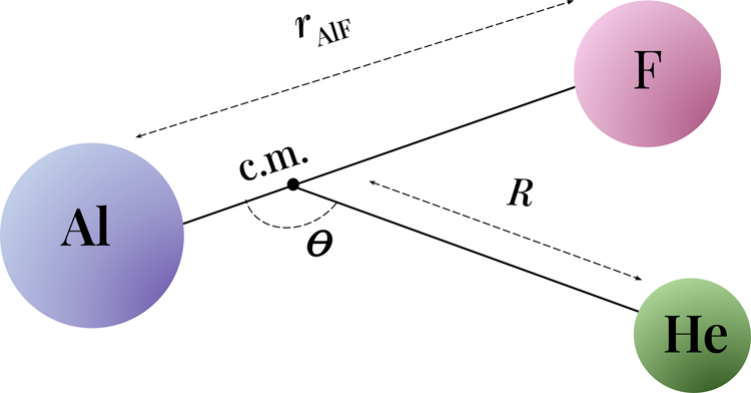
Jacobi coordinates for the AlF + He system.

The explicitly correlated coupled cluster method^34^ restricted to a single, double, and noniterative triple excitations (CCSD­(T)-F12b),^35^ is used to calculate the potential energy surfaces for the *X* ^1^ *A*′, *a* ^3^ *A*″, and *b* ^3^ *A*′ electronic states of the AlF + He complex. In CCSD­(T)-F12b computations, we use aug-cc-pV6Z^36^ as the orbital basis set, aug-cc-pV6Z-RIFIT as the density fitting and resolution of the identity basis set, and aug-cc-pV5Z-JKFIT as the density fitting basis set for the exchange and Fock operators. We obtain aug-cc-pV5Z-JKFIT by augmenting cc-pV5Z-JKFIT.^37^ cc-pV5Z-JKFIT for He is based on the unpublished work.^38^ aug-cc-pV6Z-RIFIT is based on unpublished work available in the Basis Set Exchange repository.^39–41^ We improve the description of the electronic correlation in the *X* ^1^ *A*′, *a* ^3^ *A*″, and *b* ^3^ *A*′ states by including the full triple correction

2 $$\delta V_{\mathrm{int}}^{\mathrm{CCSDT}}=V_{\mathrm{int}}^{\mathrm{CCSDT}}-V_{\mathrm{int}}^{\mathrm{CCSD}\left(T\right)}$$

where *V*_int_^CCSDT^ and *V*_int_^CCSD(T)^ are the interaction energies calculated using the CCSDT and CCSD­(T) methods, respectively, with the aug-cc-pVTZ basis set.^42–44^

We describe *A* ^1^ *A*′ and *B* ^1^ *A*″ states of the complex with the internally contracted multiconfiguration reference configuration interaction (MRCI) method with the Davidson correction^45–47^ and the aug-cc-pV5Z basis set.^42–44^ We obtain appropriate orbitals by multistate multiconfiguration self-consistent field (MCSCF) calculations.^48–51^ The active space is composed of 8 electrons distributed over 5 orbitals in the *A*′ symmetry and 1 orbital in the *A*″ symmetry.

The potential energy surfaces *V*_int_(*R*,θ) are anisotropic and can be expanded in the basis of Legendre polynomials, *P*_λ_(cos θ), as

3 $$V_{\mathrm{int}}\left(R,\theta \right)=\sum_{\lambda =0}^{\lambda_{\max}}V_{\lambda }\left(R\right)P_{\lambda }\left(\cos\theta \right)$$

where *V*_λ_(*R*) are the expansion coefficients dependent on *R*. Potential energy surfaces *V*_int_(*R*,θ) are calculated on a two-dimensional grid of 50 points in *R* between 5 and 35 bohr and 15 points in angle θ between 0 and 180°—chosen to be the roots of the Legendre polynomial of the order of 15 to facilitate Gauss-Legendre quadrature.

At long-range, the atom + molecule potential is dominated by attractive van der Waals interactions of the form

4 $$V\left(R,\theta \right)\approx -\frac{C_{6,0}}{R^{6}}-\frac{C_{6,2}}{R^{6}}P_{2}\left(\cos\theta \right)$$

where other Legendre components have leading *C*_*n,m*_ with *n* > 6. The isotropic *C*_6,0_ coefficient is the sum of the dispersion and induction contributions. The dispersion is an intermonomer correlation effect and corresponds to the interaction of fluctuating instantaneous dipole moments. The isotropic dispersion coefficient calculated from the Casimir–Polder formula is^52^

5 $$C_{6,0}^{\mathrm{dis}}=\frac{3}{\pi }\int_{0}^{\infty }{\mathrm{\alpha ̅}}^{\mathrm{AlF}}\left(i\omega \right){\mathrm{\alpha ̅}}^{\mathrm{He}}\left(i\omega \right)d\omega$$

where α̅^*X*^ is the mean dynamic dipole polarizability of *X*. The anisotropic *C*_6,2_^dis^ coefficient is given by

6 $$C_{6,2}^{\mathrm{dis}}=\frac{1}{\pi }\int_{0}^{\infty }\Delta \alpha^{\mathrm{AlF}}\left(i\omega \right){\mathrm{\alpha ̅}}^{\mathrm{He}}\left(i\omega \right)d\omega$$

where Δα^AlF^ is the anisotropy of the polarizability of the AlF molecule defined as the difference between its parallel α_∥_^AlF^ and the perpendicular α_⊥_^AlF^ dynamic dipole polarizabilities: Δα^*AlF*^ = α_∥_^AlF^ – α_⊥_^AlF^.

For AlF in the ground state, we calculate polarizabilities using the coupled cluster polarization propagator method^52–56^ and obtain *C*_6,0_^dis^ = 18.97 *E*_*h*_ *a*_0_^6^ and *C*_6,2_^dis^ = −0.13 *E*_*h*_ *a*_0_^6^. For AlF­(*a* ^3^Π)+He, we scale the ground-state isotropic and anisotropic dispersion coefficients by the ratio of the corresponding molecular static isotropic and anisotropic polarizabilities. This approach is justified because, given the negligible probability of *X* ^1^Σ – *a* ^3^Π transition, the expressions for the dispersion coefficients are similar for the Σ and Π states.^57^ For further comparison, we calculate the dispersion coefficients for AlF­(*X* ^1^Σ) + He and AlF­(*a* ^3^Π) + He using the polarizability of AlF at imaginary frequencies, determined via the damped response approach^58^ at the Hartree–Fock level. Although this method is moderately accurate due to the lack of electron correlation, it provides *C*_6,0_^dis^ for AlF­(*X* ^1^Σ) + He consistent with coupled cluster polarization propagator results within 5% accuracy. The ratio of *C*_6,0_^dis^ for AlF­(*a* ^3^Π) + He obtained through this method to those used in our scattering calculations is 0.87. Hence, we estimate that *C*_6,0_^dis^ for AlF­(*a* ^3^Π) + He has an uncertainty of approximately 20%. All our calculations indicate that *C*_6,2_^dis^ is small; however, the sign of *C*_6,2_^dis^ is altered by electron correlation in the case of AlF­(*X* ^1^Σ) + He. Given this uncertainty, we do not fix its value in our final interaction potentials.

The induction effect is due to the polarization of one monomer due to the static field of the other monomer, in this case, AlF. The induction energy is determined by the permanent multipole moments and static polarizabilities of the monomers. The induction contribution to the *C*_6,*m*_ coefficients can be written as

7 $$C_{6,0}^{\mathrm{ind}}=C_{6,2}^{\mathrm{ind}}=\mu_{\mathrm{AlF}}^{2}{\mathrm{\alpha ̅}}^{\mathrm{He}}\left(0\right)$$

where μ_AlF_ is the permanent electric dipole moment of AlF. The static polarizability of He, α̅^He^(0), is taken from ref 59. The value of *C*_6,0(2)_^ind^ is calculated to be 0.48 *E*_*h*_ *a*_0_^6^.

All electronic structure calculations are performed with the Molpro^60–62^ and mrcc^63–67^ packages of ab initio programmes.

### 2.2 Collision Theory

The essence of buffer gas cooling is the thermalization of AlF molecules by colliding with He atoms. Thus, scattering calculations are necessary to understand the possible outcomes of cooling AlF by He. We also calculate bound states of the AlF–He van der Waals complex for the *X* ^1^Σ^+^ and *A* ^1^Π states. Due to the weakly bound and highly nonharmonic nature of such states, they are most conveniently treated in the same formalism as the scattering.

We treat the system as an atom+rigid-rotor collision. The scattering Hamiltonian can be written as

8 $$Ĥ=-\frac{\hbar^{2}}{2\mu }\nabla_{R}^{2}+\frac{\hbar^{2}{\mathrm{L̂}}^{2}}{2\mu R^{2}}+V_{\mathrm{int}}\left(R,\theta \right)+B_{0}{ĵ}^{2}$$

The first term is the component of the kinetic energy operator in the scattering coordinate *R*. The second term is the centrifugal component, where *L̂* is the rotational angular momentum of He and AlF around each other, with the quantum number *L. V*_int_(*R*,θ) is the interaction potential of the colliding systems, as calculated in the previous section, and μ is the collisional reduced mass. The states of the free molecule are eigen functions of the rotational Hamiltonian, *Ĥ*_rot_ = *B*_0_ *ĵ* ^2^, with the rotational quantum number *j* and energy *j*(*j* + 1)*B*_0_, where *B*_0_ = 0.55 cm^–1^ ^68^ is the rotational constant of AlF. We neglect the small differences in rotational constant for different electronic states.

The representation of AlF as a rigid rotor is an excellent approximation when the AlF is in its *X* ^1^Σ^+^ ground state. The excited *a* ^3^Π state is more complicated since it interacts with He on two potential surfaces, as shown in Table 1. The difference between these two potential surfaces drives spin–orbit-changing collisions, as described by Alexander.^69^ Full dynamics on these coupled surfaces, as has been done for CO + He,^70,71^ would greatly add to the complexity of the scattering calculations and is beyond the scope of this paper. Instead we consider only the lowest spin–orbit component Ω = 0, neglecting coupling to the Ω = 2 component which is almost 100 cm^–1^ away.^18^ Note that there is no coupling to Ω = 1 and that coupling between the parity-doublet states *a* ^3^Π_0_^±^ occurs only in second-order through Ω = 2 states^69^ and is thus neglected here. This results in a model of scattering which is again a simple rigid-rotor AlF interacting with He, but now on a potential that is the average of the *b* ^3^ *A*′ and *a* ^3^ *A*″ surfaces. For the *A* ^1^Π state, we calculate only bound states. In this case the parity doublet molecular states *A* ^1^Π_1_^±^ are split by only 3 MHz^18^ and directly coupled by the difference potential.^69^ The individual *A* ^1^ *A*′ and *B* ^1^ *A*″ surfaces are thus recovered over essentially all of the relevant coordinate space, and we calculate bound states on the separate surfaces, neglecting the weak coupling between them.

The expansion coefficients *V*_λ_(*R*) of the interaction potentials are interpolated and extrapolated using a reciprocal power reproducing kernel Hilbert space method (RP-RKHS).^72^ This produces potentials with asymptotic forms as a sum of terms with different inverse powers depending on the parameters used. Terms with different values of λ in the expansion have different leading terms, and we have chosen parameters in the method to give the correct leading powers up to *R* ^–10^. For the λ = 0 term, we use the method of ref 73 to fix the extrapolation to the *C*_6,0_ calculated in Section 2.1.

We perform coupled-channel scattering calculations using the molscat package.^74,75^ The angular component of the wave function is expanded in the total angular momentum basis,^76^ limited by *j*_max_ = 12. Coupled equations are propagated using the Manolopoulos diabatic modified log-derivative^77^ and the Alexander-Manolopoulos Airy propagator.^78^ The solutions are matched to asymptotic boundary conditions and *S* matrices are extracted using the usual methods. Elastic and state-to-state inelastic cross sections are given by

10 σj→j′=∫dσj→j′dΩdΩ(9)=ℏ2π(2j+1)2μEcoll∑J(2J+1)∑L,L′|δj,j′δL,L′−SjL,j′L′J|2(10)

where dΩ is an element of solid angle and dσ/dΩ is a differential cross section. Since we are interested in buffer gas cooling, the cross section of relevance is not generally the elastic cross section but instead a thermalization or momentum transfer cross section,^76,79,80^

11 $$\sigma_{\mathrm{th},j}=\int \frac{d\sigma_{j\to j}}{d\Omega }\left(1-\cos\Theta \right)d\Omega$$

where Θ is the polar scattering angle associated with Ω. This cross section is also relevant to diffusion.^81^ It differs from the elastic cross section by interference terms between scattering for *J,L* and *J* ± 1, *L* ± 1. The explicit expressions in terms of the S matrix are tedious but readily evaluated and are given by Arthurs and Dalgarno.^76^ The thermally averaged elastic and inelastic cross sections are given by

12 $$\left\langle \sigma \right\rangle =\int_{x=0}^{\infty }xe^{-x}\sigma \left(E_{\mathrm{coll}}\right)dx$$

where *x* = *E*_coll_/*k*_B_ *T*. The thermalization cross section requires an additional factor of *x*/2 in the integrand, since higher energy collisions lead to more thermalization.

Bound states are calculated using the bound package.^75,82^ This is closely related to molscat and uses similar coupled-channels methods, but matches to bound-state boundary conditions and solves for eigenenergies.^83^

## 3 Results and Discussion

### 3.1 Potential Energy Surfaces

The equilibrium geometries and corresponding well depths for the studied electronic states of AlF + He are collected in Table 2. The calculated well depths for global minima range from 21 to 28 cm^–1^, except for the *a* ^3^ *A*″ state. They are only slightly deeper than that of a comparable YbF + He complex (21.88 cm^–1^ ^84^) and lie on the lower end of the typical interaction strength scale for interactions between neutral molecules and He.^85^ The *a* ^3^ *A*″ state is a notable exception, with a potential well depth approximately twice as deep, placing it near the higher end of the He + neutral molecule interaction strength scale.^85^ This significant difference indicates that the interaction strength between AlF and He can be controlled by electronic excitation.

**Table 2:** Equilibrium Geometries *R*_*e*_, *θ*_*e*_ and Equilibrium Well Depths *D*_*e*_ of the AlF + He Complex in Different Electronic States^a^

state	minima	*R*_*e*_ (bohr)	θ_*e*_ (degree)	*D*_*e*_ (cm^–1^)
*X* ^1^ *A*′	gm	7.71	180	24.9
		7.1 ^17^	180 ^17^	22 ^17^
		7.75 ^30^	180 ^30^	24.056 ^30^
	lm	10.06	0	8.0
*b* ^3^ *A*′	gm	7.17	141.4	27.5
	lm	8.68	0	21.6
	lm	7.59	180	27.2
*a* ^3^ *A*″	gm	6.00	85.4	54.1
	lm	7.59	180	27.2
*A* ^1^ *A*′	gm	7.62	180	24.0
	lm	9.05	0	22.1
*B* ^1^ *A*″	gm	7.62	180	24.0
	lm	9.05	0	22.1

a Values for global (gm) and local (lm) minima are reported.

Figure 2 presents one-dimensional cuts through the potential energy surfaces for the *X* ^1^ *A*′, *a* ^3^ *A*″, *b* ^3^ *A*′, *A* ^1^ *A*′, and *B* ^1^ *A*″ electronic states of AlF interacting with He in two linear configurations (θ = 0 and 180°) and one perpendicular configuration (90°). For the ground state of AlF (Figure 2(a)), the linear configuration with helium near fluorine displays a potential well that is twice as deep as that of both the perpendicular and alternate linear configurations, occurring at a significantly larger intermonomer separation. The *X* ^1^Σ^+^ → *a* ^3^Π and *X* ^1^Σ^+^ → *A* ^1^Π electronic excitations of AlF are dominated by the transition of one electron between 3s and 3p orbitals localized on Al atom, and mostly affects the electronic density of Al.^16^ Thus, the potential energy curves for θ = 180° are similar for all electronic states. Electronic excitation of AlF allows helium to come closer to aluminum and reduces the differences in the well depths for two linear configurations. Additionally, the geometry of global minima is strongly affected by electronic excitation (see next paragraphs). This illustrates how the shape of the interaction potential can be controlled by varying the electronic state of AlF.

**Figure 2:**
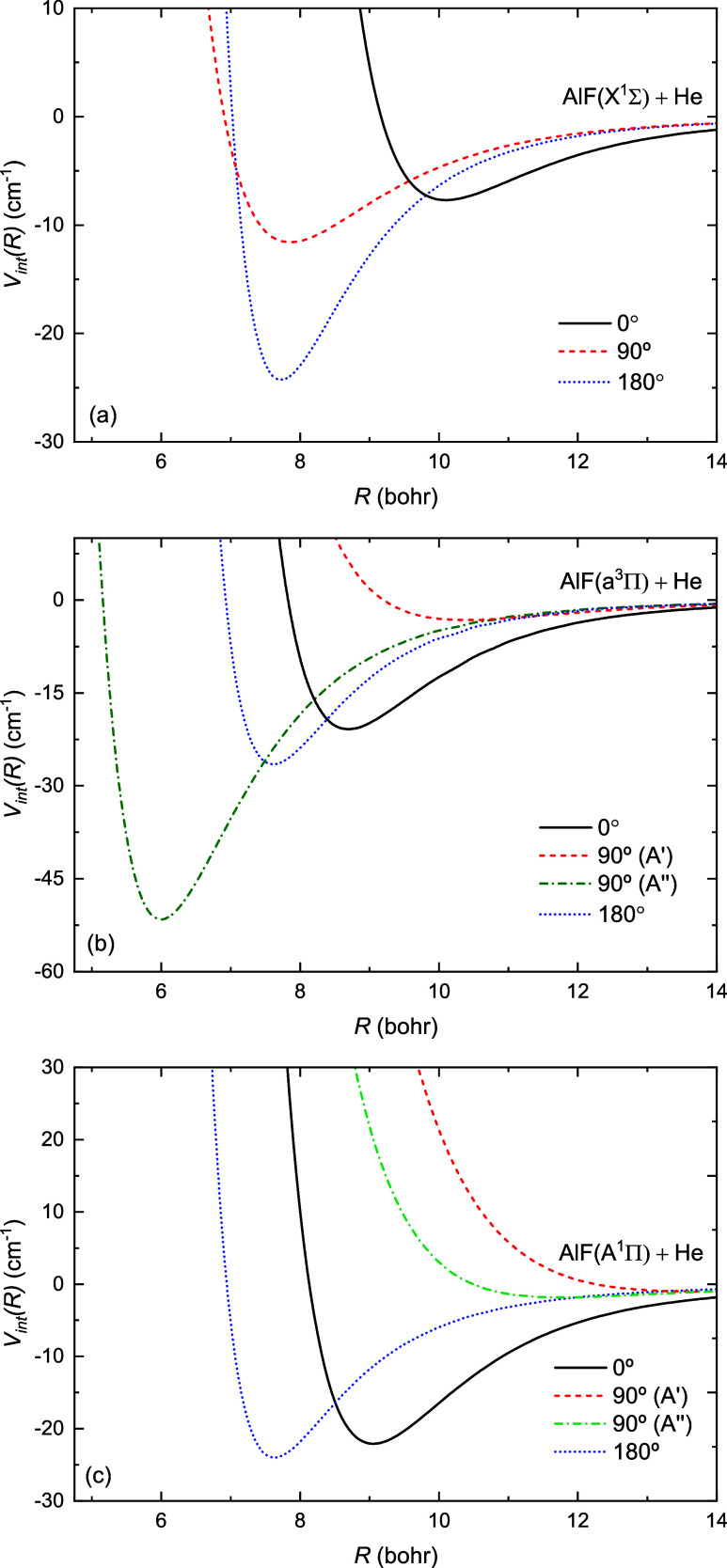
One-dimensional cuts through the potential energy surfaces for the (a) *X* ^1^ *A*′, (b) *a* ^3^ *A*″ and *b* ^3^ *A*′, and (c) *A* ^1^ *A*′ and *B* ^1^ *A*″ electronic states of AlF + He in linear and perpendicular orientations.

Figures 3 and 4 show two-dimensional contour plots and corresponding Legendre components of the interaction potentials for the *X* ^1^ *A*′, *a* ^3^ *A*″, *b* ^3^ *A*′, *A* ^1^ *A*′, and *B* ^1^ *A*″ electronic states of AlF + He. The anisotropic nature of the studied interatomic interactions is clearly visible, with all surfaces showing a strong orientation dependence. The minimum of the *X* ^1^ *A*′ state has the He interacting with the more polarizable F end of the AlF molecule (θ = 180°). The interaction in this region of the potential is relatively insensitive (within 10%) to excitation of the molecule, which is consistent with the excitations being dominated by transitions centered on the Al atom. For the *b* ^3^ *A*′ state (Figure 3(b)), the minimum is distorted away from θ = 180° to a bent configuration, but the shape is similar. However, when the He approaches AlF perpendicular to the molecular axis, the *b* ^3^ *A*′ shows significant repulsion in the intermediate regime and the *a* ^3^ *A*″ shows a substantial well about 75% deeper than the linear minimum, forming the global minimum for this state at a roughly T-shaped geometry. We attribute these interactions primarily to the strong quadrupole moment of the excited *p* orbital on the Al in these excited states.

**Figure 3:**
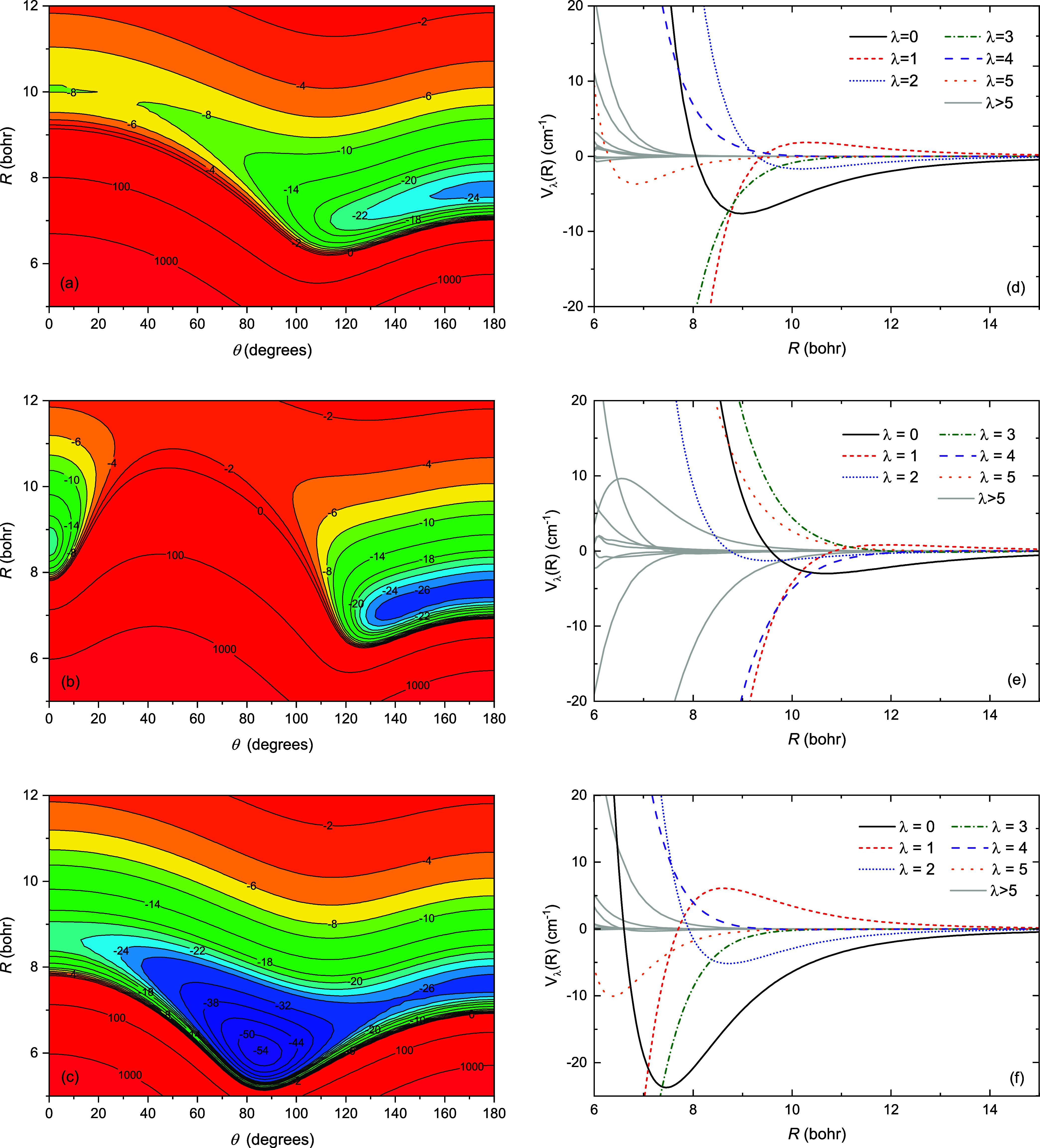
Two-dimensional potential energy surfaces and corresponding Legendre components for the (a,d) *X* ^1^ *A*′, (b,e) *b* ^3^ *A*′, and (c,f) *a* ^3^ *A*″ electronic states of AlF + He.

**Figure 4:**
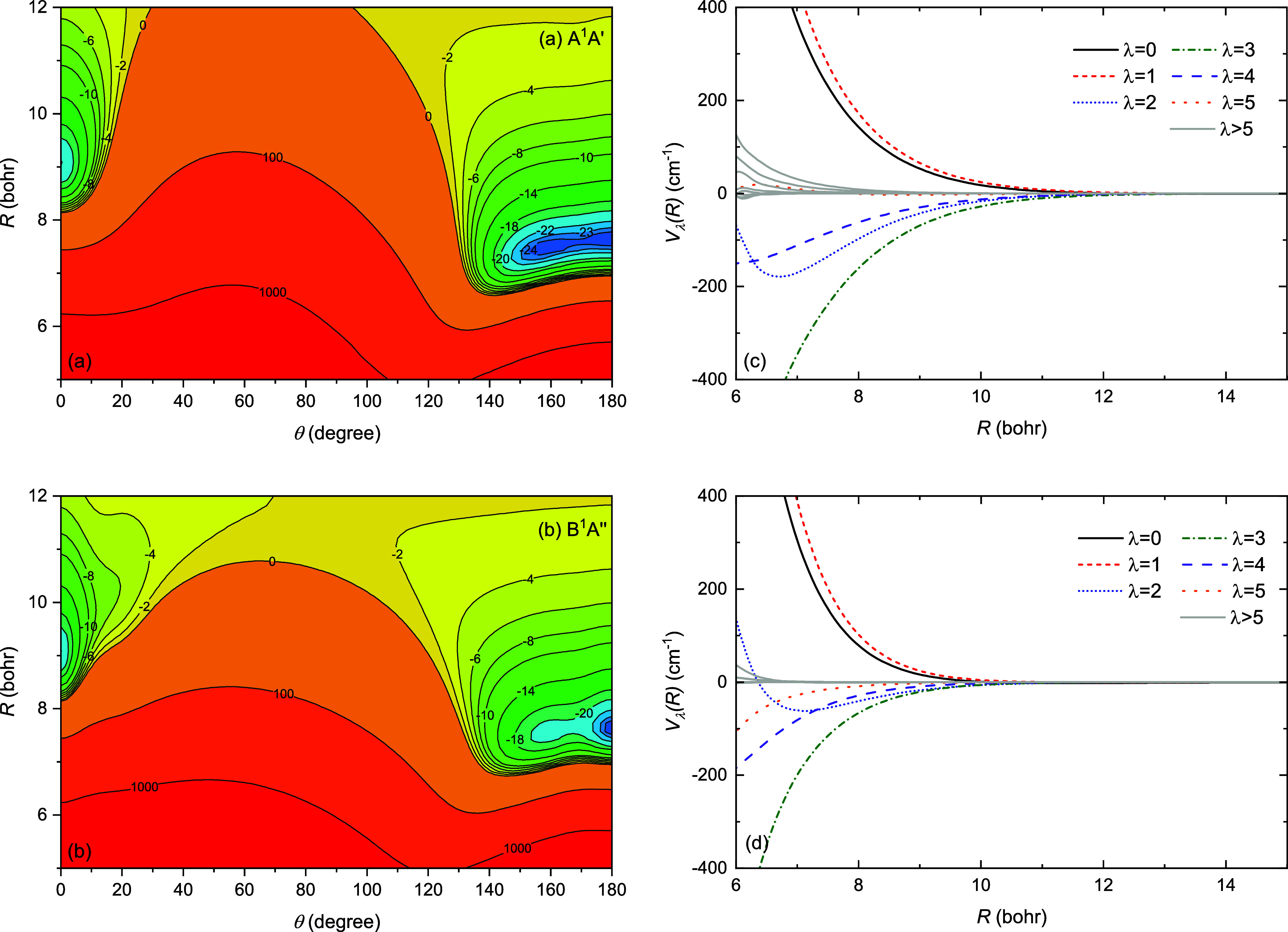
Two-dimensional potential energy surfaces and corresponding Legendre components for the (a,c) *A* ^1^ *A*′ and (b,d) *B* ^1^ *A*″ electronic states of AlF + He.

For the *X* ^1^ *A*′, *b* ^3^ *A*′, *A* ^1^ *A*′, and *B* ^1^ *A*″ states, there is a local minimum in the linear configuration where He reaches the molecule from the side of Al. The depths of these minima are approximately 8, 21, 22.1 and 22.1 cm^–1^ for *X* ^1^ *A*′, *b* ^3^ *A*′, *A* ^1^ *A*′ and *B* ^1^ *A*″ states, respectively. For the *a* ^3^ *A*″ state, this feature also exists, but is not a minimum as it joins up with the T-shaped global minimum. This local minimum for the *X* ^1^ *A*′ state was not reported in the previous studies,^17,30^ as it only emerges after including the full triple correction. This minimum is quite shallow, with the barrier between global and local minima having energy only a fraction of cm^–1^ higher than the local minimum. The existence of this minimum for the excited states can also be attributed to the quadrupole on the Al atom. The examination of the potential energy surfaces provides insights into the nature of interactions within the AlF + He complex.

The accuracy of our electronic structure calculations may be affected by (a) an incomplete orbital basis set, (b) an inadequate description of the correlation energy, and (c) the neglect of relativistic effects.^86,87^ Basis sets with increasing cardinal numbers are used to study convergence toward the complete basis limit. As shown in Figure 5(b), the interaction strength for the *X* ^1^ *A*′ state increases monotonically as the basis set size increases. The difference in the well depth of the potentials predicted by CCSD­(T)-F12 using aug-cc-pV5Z and aug-cc-pV6Z basis sets is 0.28 cm^–1^. For sextuple zeta basis sets, the difference between CCSD­(T) and CCSD­(T)-F12 is about 0.5 cm^–1^. The complete basis set limit estimated using CCSD­(T) is deeper by 0.3 cm^–1^ than the depth of the well depth estimated by CCSD­(T)-F12.

**Figure 5:**
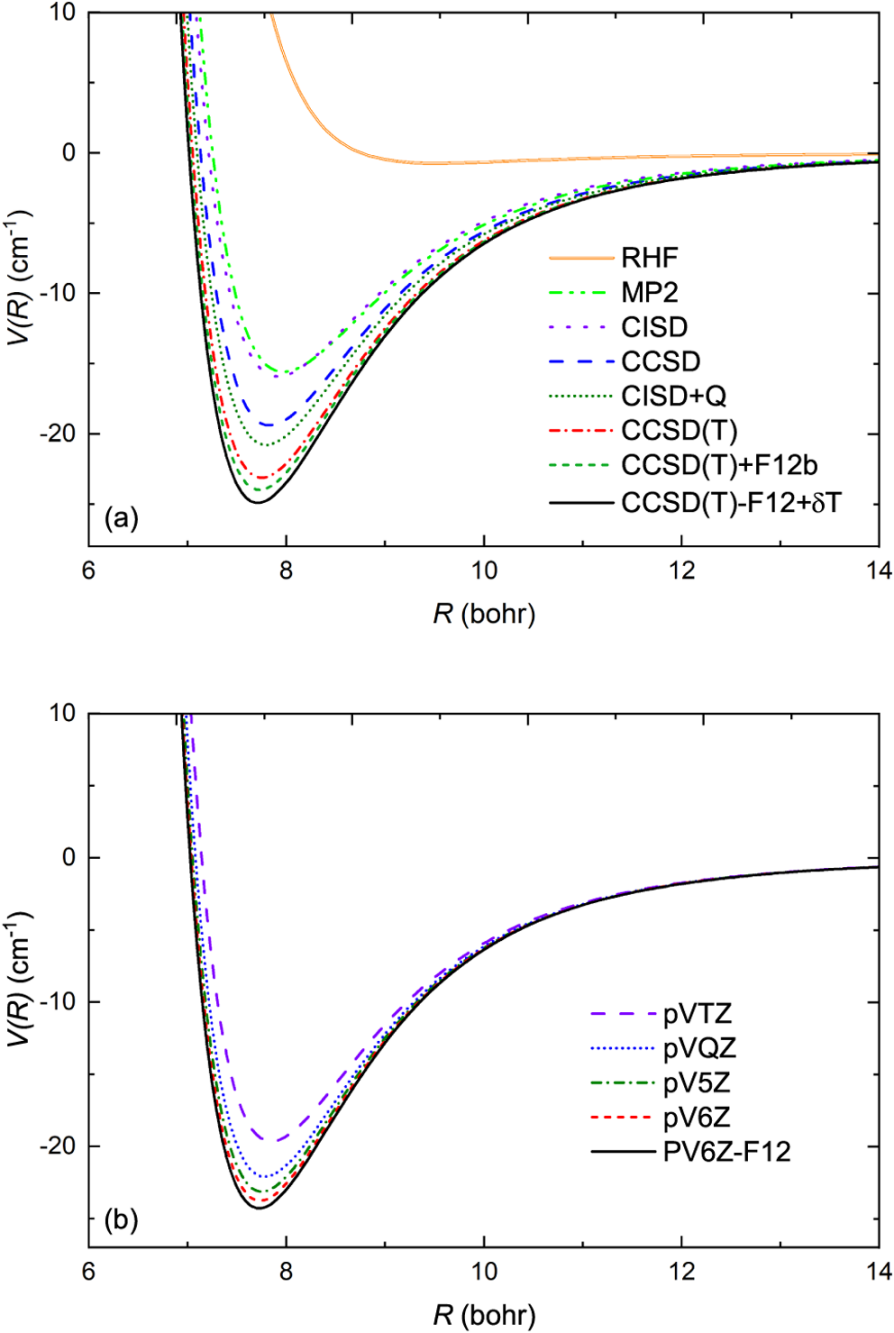
Convergence of the interaction energy of AlF + He in the *X* ^1^ *A*′ state at θ = 180° (a) for different ab initio methods using the aug-cc-pV5Z basis set and (b) for the aug-cc-pV*X*Z basis set with increasing cardinal number *X* using the CCSD­(T) method.

The correct description of the electron correlation is crucial for AlF + He, similar to other systems dominated by the van der Waals interaction. The convergence of interaction energy for the *X* ^1^ *A*′ state with the quality of the wave function is analyzed in Figure 5(a). The Hartree–Fock method, which is a mean-field method, produces a repulsive potential. The second-order perturbation theory, MP2, reproduces about 75% of the correlation energy. The depth of the potential predicted at the CCSD level is 19.4 cm^–1^. Further perturbative inclusion of triple excitation deepens the potential by 3.7 cm^–1^ while the full triple correction improves it further by 0.62 cm^–1^. The perturbative treatment of the triple excitation is responsible for approximately 85% of the total triple contribution. When calculating the entire potential energy surface, we neglect the excitations higher than triple in the coupled cluster expansion. We estimate that contribution as a difference between CCSDT­(Q) and CCSDT potential, and it turns out to be relatively small, as presented in Figure 6. It alters the interaction energy by 0.5 cm^–1^ near the classical turning point and decreases rapidly with the intermonomer distance to more than ten times lower value (0.045 cm^–1^) at the global minimum. The reported potential energy surface also neglects the core and core–valence correlation. Including the core and core–valence correlation changes the interaction energy by 0.02 cm^–1^ at the equilibrium geometry, as we estimate at the CCSD­(T) level of theory.

**Figure 6:**
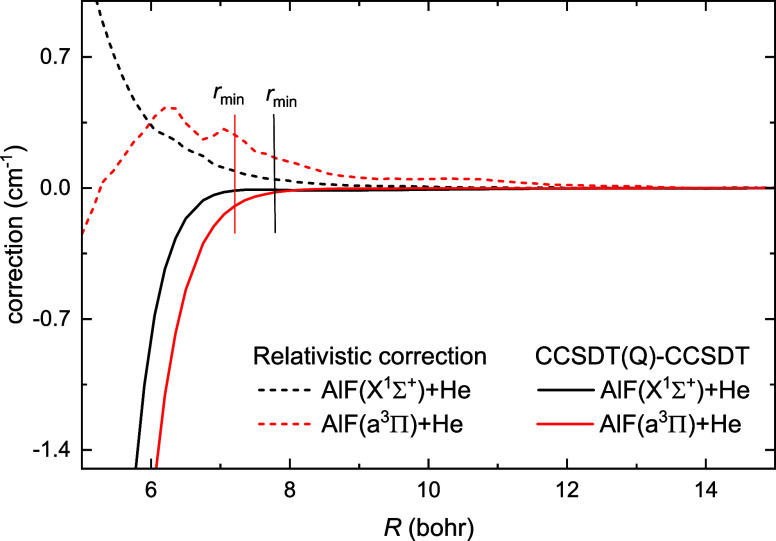
Effect of including noniterative quadruple excitations within the CCSDT­(Q) method and the relativistic correction within the Douglas–Kroll–Hess Hamiltonian on the interaction energy of AlF + He at θ = 180° in *X* ^1^ *A*′ (black) and degenerate *b* ^3^ *A*′ and *a* ^3^ *A*″ (red) states. The vertical lines show the position of the minima for the corresponding potentials.

Since the system under investigation consists of light atoms, relativistic effects are small. This can be seen in the CCSD­(T)/aug-cc-pV5Z calculations with the Douglas-Kroll-Hess Hamiltonian included up to the third order presented in Figure 6. The relativistic corrections for *X* ^1^ *A*′ and *b* ^3^ *A*′ are respectively 0.05 and 0.3 cm^–1^ around the global minimum and decrease with increasing intermonomer distance.

Finally, we test the accuracy of the rigid rotor approximation by optimizing the structures of AlF (*X* ^1^Σ^+^), and AlF (*X* ^1^Σ^+^) + He using CCSD­(T)/aug-cc-pV5Z. The interaction with He decreases the equilibrium bond length of AlF by 0.008 bohr. This small relaxation of the bond length changes the interaction energies by a negligible 0.02 cm^–1^.

The accuracy of our calculations is predominantly limited by the basis set incompleteness in the description of the valence electron correlation. The overall uncertainty of our potential at the global minimum is estimated to be 0.3 cm^–1^, which is 1.3% of the well depth of the ground state potential. Our ground state interaction potential exhibits well depth similar to that in ref 30, but it is deeper by 2.9 cm^–1^ (12%) than that reported in ref 17. This discrepancy is not surprising, as the calculations reported in ref 17 do not reach the complete basis set limit. The older results reported in ref 30 used the midbond functions, which improve the convergence of the interaction energy calculations with the basis set size. The basis set incompleteness hampers the accuracy of our potential in the *b* ^3^ *A*′ state by 1 cm^–1^, which is 3.9% of the well depth of the *b* ^3^ *A*′ potential and 2% of the well depth of the *a* ^3^ *A*″ potential, the same for the *A* ^1^ *A*″ and *B* ^1^ *A*′ state (MRCI/aug-cc-pV5Z) is 0.4 cm^–1^ which is 1.6% of the depth of the potential. We expect that the MRCI computations are affected mainly by the lack of higher excitations in the configuration interaction expansion and the size inconsistency of the method. We do not have a good estimation of those contributions. Still, we may expect that it can be even of the order of 10–20%, similar to the difference between the CCSD and CCSDT interaction energies in the ground state.

### 3.2 Collision Dynamics

When AlF collides with He, there are two possible outcomes: elastic collision, where the internal state of AlF is conserved, and inelastic collision, where the internal state changes. Elastic collisions determine the translational thermalization of AlF, weighted by scattering angle to give the thermalization cross section σ_tr_ as discussed above, whereas rotational thermalization is determined by the inelastic cross section. Figure 7 shows the thermalization and inelastic cross sections for AlF in its *X* ^1^Σ^+^ and *a* ^3^Π_0_ states colliding with He; the corresponding elastic cross sections are given in Supporting Information. To better understand these outcomes, we can examine the cross sections of these events.

**Figure 7:**
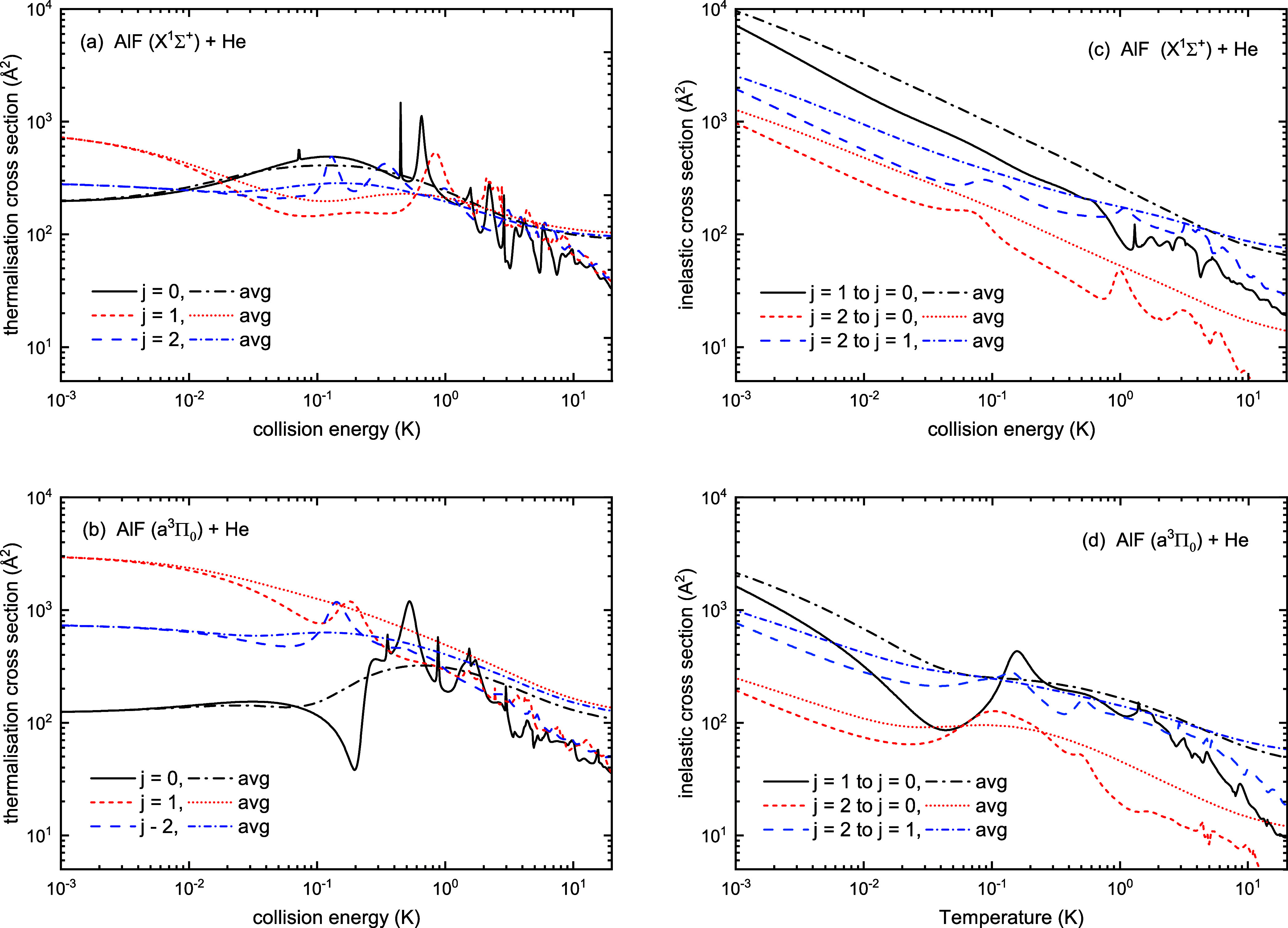
Thermalization and inelastic cross sections as a function of the collision energy for AlF + He scattering in the (a,c) *X* ^1^Σ^+^ and (b,d) *a* ^3^Π_0_ states of AlF. The cross sections of the scattering channels for the rotational states *j* = 0,1,2 of AlF are shown. The corresponding curves in the same color are the thermally averaged cross sections.

Let us focus on the temperatures between 100 mK to 10 K; this is the most relevant range because 100 mK is the lowest temperature for which buffer gas cooling is typically effective. The values of the ratio of thermalization and inelastic cross sections at 1 K are reported in Table 3 for scattering of He with AlF in the *X* ^1^Σ^+^ and *a* ^3^Π_0_ states.

**Table 3:** Ratio of Thermally Averaged Thermalization Cross Section to Inelastic Cross Section, ⟨*σ*_th,*j*_⟩/⟨*σ*_*j*→*j*′_⟩, and Thermally Averaged Rotationally Inelastic State-to-State Collision Cross Section (in Å^2^), ⟨*σ*_*j*→*j*′_⟩, for AlF + He Collisions at 1 K

	ratio			rotationally inelastic		
state	⟨*σ*_th,0_⟩/⟨σ_1→0_⟩	⟨*σ*_th,0_⟩/⟨σ_2→0_⟩	⟨*σ*_th,1_⟩/⟨σ_2→1_⟩	⟨σ_1→0_⟩	⟨σ_2→0_⟩	⟨σ_2→1_⟩
*X* ^1^Σ^+^ + He	0.92	0.81	1.11	253.79	51.03	172.26
*a* ^3^Π_0_ + He	2.58	15.85	4.16	118.18	19.30	114.57

A significant value of σ_th_ is necessary to provide effective translational thermalization. The exact value required depends on the experimental setup, but 100 Å^2^ is a typical benchmark.^11,12^ The cross sections for both molecular states considered are above this over the whole range presented here, with little difference between rotational states at 1 K and above. However, to lower energy the cross section for the *j* = 0 states for both *X* ^1^Σ^+^ and *a* ^3^Π_0_ are the lowest, barely above 100 Å^2^. There is a significant dip in σ_th_ for *a* ^3^Π_0_ at 0.2 K. This is a signature of a Feshbach resonance causing the phase shift to pass through 0; the corresponding peak is only partly resolved due to increasing contributions from higher partial waves. However, this feature is narrow enough that it has only a small effect on the thermally averaged cross section. It is therefore likely that translational thermalization of these states will be possible, but may be less efficient at reaching the lowest temperatures for setups with particularly short extraction times or small buffer-gas cells. Nonetheless, these cross sections are comparable to those measured experimentally for He + CaH,^88^ although somewhat smaller than those calculated for the same system.^89^

Rotational thermalization relies on the inelastic cross sections. These are compared to the elastic (or thermalization) cross section, and a ratio of 1:10 or 1:100 is usually recommended for efficient rotational thermalization, since translational thermalization typically takes 50–100 collisions.^11^ There is more variation between the rotational states for rotational relaxation than for translational thermalization, with *j* = 1 → 0 being the fastest process for both states. Nonetheless, the inelastic cross sections are all within a factor of a few of the thermalization cross sections at 1 K, and the ratio rises to lower temperatures. The rotational thermalization is thus expected to be highly efficient from all rotational levels of both *X* ^1^Σ^+^ and *a* ^3^Π_0_ electronic states. This is driven by the strongly anisotropic potentials, as shown in Figure 3, and is consistent with previous findings both for this system^17^ and other similar ones.

The bound rovibrational levels of the AlF + He complex in the X^1^ *A*′ state are reported in Table 4. The lowest bound state supported by this potential is at −7.59 cm^–1^. There are 26 bound states for the ground state in total. The bound states for the *A* ^1^ *A*′ and *B* ^1^ *A*″ potentials, correlating with the *A* ^1^Π excited state of AlF, are tabulated in Supporting Information. These have relevance for optical transitions in the AlF–He complex and may be of interest for further study.

**Table 4:** Bound Rovibrational Levels of AlF + He in the *X* ^1^ *A*′ State^a^

*J* ^*p*^					
0^+^	–7.59	–2.44	–0.63		
1^+^	–4.77	–0.03			
1^–^	–7.22	–4.84	–2.01	–0.81	
2^+^	–6.50	–4.13	–1.52	–1.16	–0.43
2^–^	–3.96	–1.45			
3^+^	–2.75	–0.41			
3^–^	–5.43	–3.07	–0.71		
4^+^	–4.01	–1.68			
4^–^	–1.16				
5^–^	–2.27				
6^+^	–0.21				

a *J* is the total angular momentum, *p* is the parity of the state. Energies are in cm^–1^.

We study the sensitivity of our scattering results to interaction energy by scaling potential by ±10% and observe changes in the elastic cross sections. Figure 8(a) shows how the cross section varies with a scaling of the potential for four energies. Here, λ scales the potential energy as *V* → (1 + λ) × *V*. We observe a peak in the 1 K line (Figure 8(a)), which can be attributed to the sensitivity of the shape resonances visible around 1 K in Figure 7(a). The uncertainty of the potential energy surface is about 2%, so the scattering results are not very far from the truth for all four energies. In Figure 8(b) we show the shift in elastic cross sections for a ± 1.5% scaling for two rotational states of AlF. For the *j* = 0 rotational state of AlF, the first few shape resonances are more sensitive to the scaling of potential, but their relative sensitivity decreases for higher energies. For the *j* = 1 rotational state of AlF, the curves are shifted while preserving the general shape. The scattering cross sections generally lie in the same range as predicted recently in ref 17, with a shift in the position of shape resonances. Although our ab initio calculations are more accurate, the scattering results from ref 17 are similar, which suggests that the details of the potential energy surface have a moderate effect on the scattering outcomes for this system.

**Figure 8:**
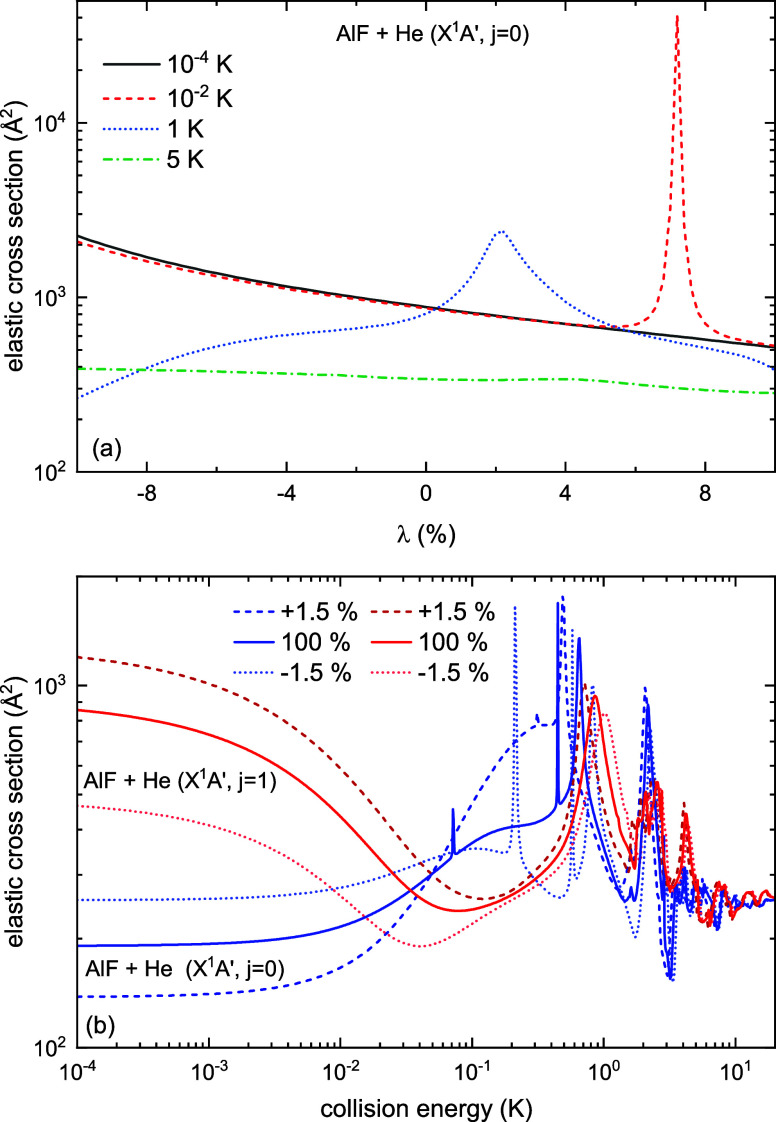
(a) Sensitivity of the elastic scattering cross section to the scaling of the potential for the *X* ^1^ *A*′ state of AlF + He for the collision energies 5, 1, 0.01, and 0.0001 K. (b) Elastic scattering cross sections as a function of the collision energy for the *X* ^1^ *A*′ potential scaled by ±1.5%.

## 4 Conclusions

We have constructed accurate potential energy surfaces using the explicitly correlated coupled-cluster and multireference configuration interaction methods for the AlF + He interactions with AlF in the ground and excited electronic states. We have found that interactions between AlF and He are weak and anisotropic. For all electronic states, we have identified global and local minima. For most of the states, the global minimum is linear and appears when He approaches AlF from the F side. The only exception from this rule is the *a* ^3^ *A*″ state, which exhibits a deeper minimum at bent geometry in addition to the linear minimum. AlF + He is a relatively light system uninfluenced by relativistic effects. We have demonstrated that the shape and depth of the AlF + He interaction potential can be controlled by changing the electronic state of AlF. The calculated potential energy surfaces are provided in numerical form in the Supporting Information.

Next, we have used coupled-channel scattering theory to calculate the elastic and inelastic scattering cross sections of the AlF + He collision outcomes. The uncertainties of the potential energy surfaces have been estimated, and the sensitivity of the scattering results to the scaling of the potential has been studied to give us a better idea of the predictability of the results. The presented data can help to determine the optimal conditions for buffer gas cooling. The calculated cross sections for the *X* ^1^Σ^+^ state are large for rotational relaxation; for translational thermalization they are moderate but still above 100 Å^2^. This indicates efficient translational and rotational cooling, in agreement with previous experiment^21^ and theory.^17^ Our calculations for the metastable state *a* ^3^Π_0_ show that cooling should also be similarly effective in this state, and that this is a promising candidate for further experiments. Further work is needed to investigate collisions with the higher spin–orbit states *a* ^3^Π_1_ and *a* ^3^Π_2_, and calculate cross sections for relaxation processes in magnetic and/or electric fields. We also calculated bound states for AlF­(*X* ^1^Σ^+^)–He which might be formed in three-body collisions in a dense cold gas, and bound states for AlF­(*A* ^1^Π)–He, which are of spectroscopic interest.

## Supplementary Material



## Notes

Tabulations of interaction potentials, elastic cross sections, and bound states (ZIP)
