# Supplementary material for: Interactions and Cold Collisions of AlF in the Ground and Excited Electronic States with He
Source: J Phys Chem A. 2025 Sep 2;129(36):8239–50. doi: 10.1021/acs.jpca.5c02533 (PMC12434673; doi:10.1021/acs.jpca.5c02533)
Supplement: Supplementary file 1 [file jp5c02533_si_001.zip › README.pdf]

Supporting Information: Interactions and Cold Collisions of AlF in the Ground and Excited Electronic States with He

Sangami Ganesan-Santhi, Matthew D. Frye, Marcin Gronowski, and Michał Tomza*

Faculty of Physics, University of Warsaw, Pasteura 5, 02-093 Warsaw, Poland

E-mail: michal.tomza@fuw.edu.pl

Potential energy surfaces

The files X1A1pes.txt, a3A2pes.txt, b3A1pes.txt, A1A2pes.txt, B1A1pes.txt contain the *ab initio* interaction energies for the X^1A', a^3A'', b^3A', A^1A', B^1A'' states of AlF +He. The interaction energies are defined by equation (1) from the main text. The interaction energy for X^1A', a^3A'', b^3A' states is at CCSD(T)-F12/aug-cc-pV6Z + (CCSDT-CCSD(T))/aug-cc-pVTZ level of theory and for the A^1A', B^1A'' is at MRCI+Q/aug-cc-pV5Z level of theory. The first column in the above files is the distance between He and the centre of mass of AlF, R. The first row is the angle between R and the molecular axis, θ. The energies are in cm^{-1}, R in bohr and θ in degree.

Collision cross section

The files X1Sigmathcs.txt, a3PiHethcs.txt contain the thermalization cross sections and thermally averaged thermalization cross sections for the $X^1\Sigma^+ + \mathrm{He}$ and $a^3\Pi_0 + \mathrm{He}$ states, respectively. The files X1Sigmaelcs.txt and a3PiHeelcs.txt contain the corresponding elastic cross sections, and the files X1Sigmainelcs.txt and a3PiHeinelcs.txt contain the corresponding inelastic cross sections. The format of the files is as follows. The first column indicates the collision energy in K $\times$ k_B, the second, third and fourth columns indicate thermalisation/elastic/inelastic cross sections in units of Å^2, for the rotational levels $j = 0, 1, 2$ (thermalisation/elastic) or $j = 1 \to 0$, $j = 2 \to 1$, and $j = 2 \to 0$ (inelastic). The fourth column indicates temperature in K followed by the corresponding thermally averaged thermalisation/elastic/inelastic cross section in Å^2.

Bound states

The file boundstates.txt contains the bound states of the AlF+He complex in the a^3A'', b^3A', A^1A' and B^1A'' states. The first column indicates the total angular momentum and

the parity of the states as J_{tot}, p, the second column indicates the ascending order of states. The energies are in cm^{-1}.
